# Editorial: Next-generation radiotherapy for prostate cancer: precision, personalization, and technological advances

**DOI:** 10.3389/fonc.2026.1994824

**Published:** 2026-10-13

**Authors:** Giuseppe Carlo Iorio, Silvia Morbelli, Marco Oderda, Alan Dal Pra, Umberto Ricardi

**Affiliations:** 1 Department of Oncology, School of Medicine, University of Turin, Torino, Italy; 2 Department of Nuclear Medicine, AOU Città della Salute e della Scienza di Torino, University of Turin, Turin, Italy; 3 Urology Clinic, Department of Surgical Sciences, City of Health and Sciences at Molinette University Hospital, University of Turin, Turin, Italy; 4 Department of Radiation Oncology, University of Miami, Miami, FL, United States

**Keywords:** modern radiotherapy, molecular imaging, precision oncology, prostate cancer, PSMA, RT, SBRT, treatment personalization

Radiotherapy (RT) plays a central role across the prostate cancer (PCa) disease continuum. Over the past decade, advances in treatment delivery, image guidance, molecular imaging, genomic biomarkers and artificial intelligence, together with the increasing adoption of hypofractionation and metastasis-directed therapy (MDT), have progressively transformed the role of radiation oncology in PCa. These developments are enabling shorter, more precise, and increasingly personalized treatments across localized, recurrent, and metastatic disease. This Research Topic brings together 12 articles exploring contemporary advances in PCa RT, encompassing both clinical evidence and technological innovation.

Among the most significant advances in radical radiotherapy for localized PCa, treatment shortening through ultra-hypofractionation has progressively reshaped contemporary practice. The randomized PACE-B trial established the non-inferiority of five-fraction stereotactic body RT (SBRT) compared with conventionally or moderately hypofractionated RT (1), while the recently reported 10-year results of HYPO-RT-PC further strengthened the long-term evidence supporting ultra-hypofractionation (2). Beyond treatment shortening, increasing attention has focused on selective dose intensification to the dominant intraprostatic lesion. The FLAME trial demonstrated a sustained biochemical control benefit from focal boosting (3), while HYPO-FLAME explored the integration of this concept within an ultra-hypofractionated approach (4).

In this Research Topic, Hands et al. demonstrated the feasibility and favorable preliminary toxicity profile of focal boosting to 84 Gy within a 28-fraction regimen, whereas Bhargava et al. reported acceptable urinary quality-of-life outcomes following prostate SBRT with intraprostatic boost. Technological innovation is also expanding the potential for more precise treatment delivery. Yacoub et al. comprehensively reviewed the evolution of proton therapy in PCa, highlighting its potential for reducing integral dose and improving normal-tissue sparing, particularly in complex treatment volumes. Extending the particle-therapy perspective, Kim et al. showed that hydrogel spacer insertion substantially reduced rectal exposure and improved plan robustness in patients undergoing carbon-ion radiotherapy. Treatment optimization may also increasingly involve reconsideration of traditional preparation protocols: Choi et al. developed a deep learning-based dose-prediction framework supporting efficient and personalized planning within an empty-bladder protocol at Mayo Clinic. Finally, Malygina et al. demonstrated that daily online adaptation significantly improved target coverage, conformity and dose homogeneity compared with non-adapted plans, without compromising organs-at-risk sparing.

In the postoperative setting, contemporary research has progressively sought to improve both treatment precision and patient tolerance. Consensus recommendations have progressively refined prostate-bed target delineation (5), while the phase III NRG-GU003 trial demonstrated that a shorter, moderately hypofractionated postoperative radiotherapy course can be delivered without compromising late patient-reported gastrointestinal and genitourinary outcomes (6). Within this framework, *Stewart* et al. reported the preliminary results of the randomized multicentre POPS trial, supported by the NIHR Biomedical Research Center at the Royal Marsden NHS Foundation Trust and the Institute of Cancer Research, evaluating ProSpare^®^, a self-inserted rectal obturator designed to facilitate daily image guidance during prostate-bed radiotherapy. ProSpare^®^-guided treatment using a reduced posterior margin improved rectal and anal canal dosimetry and was associated with lower acute bowel toxicity, showing very favorable patient-reported tolerability.

The recurrent setting represents another rapidly evolving field, shaped by molecular imaging and metastasis-directed approaches. Prostate-specific membrane antigen (PSMA) positron emission tomography (PET) has substantially improved disease localization at low PSA levels, challenging conventional paradigms of salvage radiotherapy and enabling individualized treatment strategies. Prospective evidence from the PSMA-SRT and EMPIRE trials has demonstrated the impact of molecular imaging on post-prostatectomy radiotherapy decision-making and provided emerging evidence that imaging-guided treatment adaptation and intensification may translate into improved oncological outcomes (7–9). In this Research Topic, Iorio et al. evaluated PSMA-PET-guided management in patients with biochemical recurrence after prostatectomy, reporting substantial treatment modification among PSMA-positive patients, including adaptation of prostate-bed radiotherapy, metastasis-directed SBRT and systemic therapy.

Beyond post-prostatectomy salvage, molecular imaging is also redefining the oligorecurrent state and facilitating more tailored MDT. The PRECISE-MDT cohort demonstrated improved outcomes with PSMA PET–guided MDT compared with choline PET–guided MDT (10–12). Consistent with these findings, Grün et al. reported high rates of local control after PSMA PET–guided SBRT for nodal and osseous oligorecurrences, with repeated courses of SBRT deferring systemic therapy in selected patients.

The role of radiotherapy is also expanding in *de novo* metastatic PCa. As evidence supporting treatment of the primary tumor in selected patients has grown, reinforced by PEACE-1 (13), interest has shifted toward comprehensive treatment strategies targeting both the primary and metastatic disease. Trials such as SOLAR are prospectively investigating this “total eradication” approach (14). Within this emerging framework, Xu et al. reported encouraging survival outcomes with definitive radiotherapy to both the prostate and all metastatic lesions in patients with oligometastatic PCa, with manageable toxicity.

Finally, advances in imaging have become integral to the increasing personalization of PCa management. PSMA-PET has substantially improved staging accuracy compared with conventional imaging, as shown by the randomized proPSMA trial (15), and its superior detection of early biochemical recurrence has further expanded its role across the disease continuum (16, 17). Beyond conventional staging and restaging indications, emerging evidence suggests additional applications for PSMA-based imaging. Liu et al. investigated the predictive value of PSMA-PET for pathological upgrading, demonstrating moderate diagnostic accuracy and underscoring its utility in pretreatment risk stratification. In the radiorecurrent setting, Merani et al. evaluated the complementary roles of multiparametric magnetic resonance imaging (mpMRI) and PSMA-PET, emphasizing that their combination may improve localization of intraprostatic recurrences, detection of extraprostatic disease, and selection of patients for local salvage strategies.

Taken together, the contributions to this Research Topic illustrate how PCa radiotherapy is evolving much beyond precision in dose delivery toward a genuinely personalized treatment paradigm. Advances in fractionation, focal dose escalation, adaptive and particle therapy, molecular imaging, and metastasis-directed approaches are progressively expanding the therapeutic scope of radiation oncology across the PCa disease continuum.
